# Simultaneous disseminated infections with intracellular pathogens: an intriguing case report of adult-onset immunodeficiency with anti-interferon-gamma autoantibodies

**DOI:** 10.1186/s12879-020-05553-y

**Published:** 2020-11-11

**Authors:** Malte Roerden, Rainer Döffinger, Gabriela Barcenas-Morales, Stephan Forchhammer, Stefanie Döbele, Christoph P. Berg

**Affiliations:** 1 Department of Hematology, Oncology, Clinical Immunology and Rheumatology, University Hospital Tübingen, Tübingen, Germany; 2 Department of Clinical Biochemistry and Immunology, Addenbrookes Hospital, Cambridge, UK; 3 Laboratorio de Inmunologia, FES-Cuautitlan, UNAM, Mexico City, Mexico; 4 Department of Dermatology, University Hospital Tübingen, Tübingen, Germany; 5 Department of Gastroenterology, Gastrointestinal Oncology, Hepatology, Infectious Diseases and Geriatrics, University Hospital Tübingen, Tübingen, Germany

**Keywords:** Non-tuberculous mycobacteria, Interferon gamma autoantibodies, Interferon gamma, Interleukin 12, Non-typhoidal salmonella, cytomegalovirus, Rituximab

## Abstract

**Background:**

Severe and disseminated non-tuberculous mycobacterial (NTM) infections are frequently linked to a genetic predisposition but acquired defects of the interferon gamma (IFNγ) / interleukin 12 (IL-12) pathway need to be considered in adult patients with persistent or recurrent infections. Neutralizing anti-IFNγ autoantibodies disrupting IFNγ signalling have been identified as the cause of a severe and unique acquired immunodeficiency syndrome with increased susceptibility to NTM and other intracellular pathogens.

**Case presentation:**

An adult Asian female with a previous history of recurrent NTM infections presented with persistent diarrhea, abdominal pain, night sweats and weight loss. Severe colitis due to a simultaneous infection with *cytomegalovirus* (CMV) and *Salmonella typhimurium* was diagnosed, with both pathogens also detectable in blood samples. Imaging studies further revealed thoracic as well as abdominal lymphadenopathy and a disseminated *Mycobacterium intracellulare* infection was diagnosed after a lymph node biopsy. Further diagnostics revealed the presence of high-titer neutralizing anti-IFNγ autoantibodies, allowing for the diagnosis of adult-onset immunodeficiency with anti-IFNγ autoantibodies (AIIA).

**Conclusions:**

We here present a severe case of acquired immunodeficiency with anti-IFNγ autoantibodies with simultaneous, disseminated infections with both viral and microbial pathogens. The case illustrates how the diagnosis can cause considerable difficulties and is often delayed due to unusual presentations. Histological studies in our patient give further insight into the pathophysiological significance of impaired IFNγ signalling. B-cell-depleting therapy with rituximab offers a targeted treatment approach in AIIA.

## Background

Th1 helper cell secretion of interferon gamma (IFNγ) is of pivotal significance for the activation of monocytes to establish an effective host defense against intracellular pathogens [1, 2]. Genetic defects of IFNγ signaling (e.g. affecting the IFNγ receptor or the transcription factor STAT1 [3–7]) thus lead to congenital immunodeficiency syndromes [1, 3] with susceptibility to infections with intracellular pathogens, particularly mycobacteria [6, 8, 9]. In *human immunodeficiency virus* (HIV)-uninfected adults however, an acquired susceptibility to intracellular pathogens is rare. In 2004, high-titer neutralizing autoantibodies against IFNγ were first identified as the cause of an acquired immunodeficiency syndrome subsequently termed *adult-onset immunodeficiency with anti-interferon-gamma autoantibodies (AIIA)* [8, 10–12]. This syndrome is characterized by a disturbed IFNγ signalling pathway leading to recurrent and disseminated infections with *non-tuberculous mycobacteria* (NTM), *non-typhoidal salmonella*, *cytomegalovirus* (CMV), *varicella zoster virus* (VZV) and other pathogens. Diagnosis of AIIA can be challenging as it involves specific testing not routinely available and clinical presentations can be unusual. We here report a rare case of a patient presenting with the nearly complete spectrum of simultaneous infections typical for this condition, where diagnosis was delayed by a misguiding secondary finding.

## Materials and methods

Whole blood or isolated peripheral blood mononuclear cells were cultivated as previously described [13]. Cytokines were measured either using standard enzyme-linked immunosorbent assays (ELISA) according to the manufacturer’s recommendations (IFNγ; Pelikine, Sanquin, NL) or on a Luminex analyzer (IL-12; Fluorokine MAP, R + D Systems, USA and Bio-Plex, Bio-Rad, UK). Anti-IFNγ antibodies were detected by ELISA and by flow cytometric bead array (Luminex analyzer) as previously described [10, 14]. IFNγ signalling was assessed by flow cytometric analysis of phosphorylated STAT1 (pSTAT1) and IL-12 production as previously described [15].

## Case presentation

**Fig. 1:**
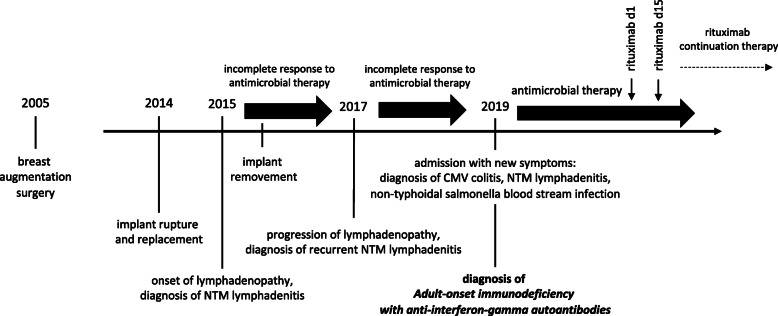
Timeline of case presentation

**Fig. 2:**
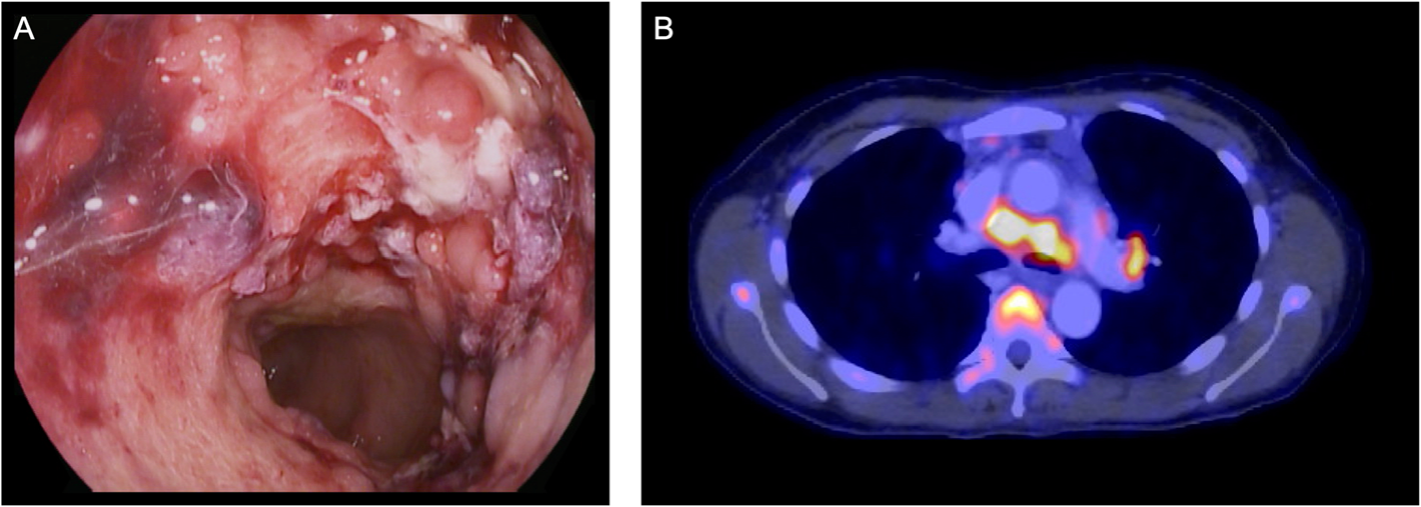
Findings in colonoscopy and PET-CT imaging. **a** Colonoscopy showing severe, ulcerating colitis due to simultaneous CMV and *Salmonella typhimurium* infections. **b** Metabolically highly active mediastinal lymphadenopathy in bioptically confirmed disseminated *Mycobacterium intracellulare* lymphadenitis

**Fig. 3:**
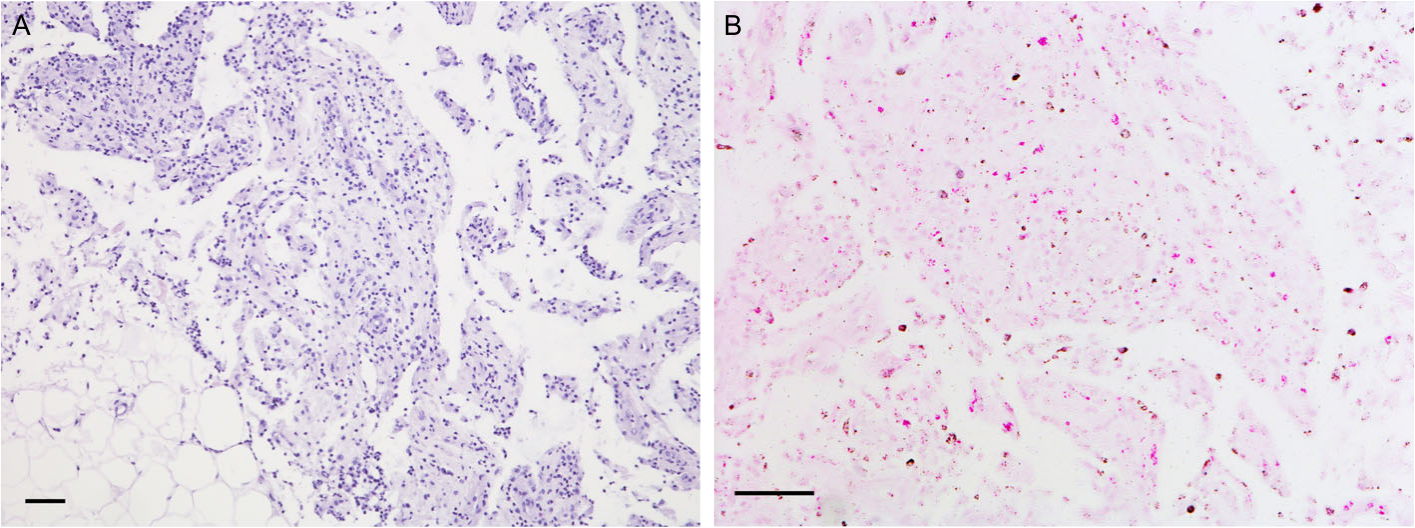
Impaired granuloma formation in a patient with IFNγ autoantibodies. **a** Skin biopsy from abdominal wall showing immature granulomas with sparse inflammatory surrounding tissue reaction. **b** Ziehl-Neelsen staining uncovers a pathogen rich mycobacterial infection. Scale bar = 100 μm

A female of 44 years and of Asian origin was admitted with fever, a two-week history of bloody diarrhea and abdominal pain. She further complained of general weakness, night sweats and weight loss. The previously healthy patient had undergone breast augmentation surgery many years before. Rupture of the breast implants had caused the formation of lymph node siliconomas and the implants had subsequently been replaced. One year after implant replacement and four years prior to presentation, the patient had first developed cervical, thoracic and axillary lymphadenopathy. NTM lymphadenitis had been diagnosed after *Mycobacterium abscessus* and *goodii* were cultured from lymph node biopsies and a connection with the silicone lymphadenopathy, assuming secondary infection had been made. Again, the implants had been surgically removed, but no mycobacteria could be cultured from intraoperative swabs or implant material. Response to the following antibiotic treatment had been slow and incomplete despite of fully drug-sensitive strains. With the new symptoms mentioned above, the patient was admitted for further diagnostic testing (timeline in Fig. 1). On admission the patient presented in a reduced general condition with fever of 39.1 °C. Persistent cervical and axillary lymphadenopathy was palpable with callous, non-tender masses. Several non-tender subcutaneous masses of up to 2 cm in diameter were also palpable alongside the abdominal wall without further skin changes present. General abdominal tenderness was notable. Stool frequency was reported as four to six times daily with bloody and mushy stools, associated with spasmodic pain. Laboratory work-up showed signs of an inflammatory process with a white blood cell count of 24,000 / μl (normal range 4100 – 11,800), a c-reactive protein of 21.1 mg/dl (normal < 0.5) and a 1 h erythrocyte sedation rate of 105 (normal range 0 – 20). Stool diagnostics revealed the simultaneous presence of a *cytomegalovirus* (CMV) as well as *Salmonella typhimurium* infection. Both pathogens were also detected in blood samples: CMV-DNA polymerase chain reaction (PCR) from EDTA blood was positive and *Salmonella typhimurium* was detected in blood cultures. Colonoscopy showed severe colitis with multiple ulcerous lesions and a biopsy confirmed CMV colitis (Fig. 2a). A positron emission tomography computed tomography (PET-CT) scan was obtained, showing both thoracic and abdominal, metabolically highly active lymphadenopathy (Fig. 2b). In line with the diagnosis of a CMV colitis, wall thickening and increased metabolic activity was present in the colon and rectum. A bronchoscopy with endobronchial ultrasound was performed to obtain diagnostic material from the enlarged mediastinal lymph nodes for further testing. While histological assessment of the lymph node biopsies showed only unspecific inflammatory changes, *Mycobacterium intracellulare* was cultured and confirmed by molecular genetics. Further, a biopsy and histological analysis of a subcutaneous lesion was performed showing acid-fast bacilli and “naked granulomas” with only sparse inflammatory surrounding tissue reaction (Fig. 3). In summary, a simultaneous disseminated NTM infection, CMV reactivation and *non-typhoidal salmonella* blood stream infection was diagnosed, prompting further diagnostics regarding an underlying immunodeficiency. Testing for HIV was negative. Peripheral blood and bone marrow flow cytometry analysis showed unremarkable populations of T and B cells and did not indicate an underlying lymphoma. Quantitative assessment of immunoglobulins revealed a polyclonal increase in IgG (2170 mg/dl, normal range 700 – 1600). On extended testing, high-titer IgG neutralizing autoantibodies against IFNγ were detected in the patient’s serum. Functional analyses demonstrated the neutralizing effect of these autoantibodies, as presence of the patient’s serum significantly impaired IFNγ signaling both in patient as well as healthy control cells, resulting in reduced induction of pSTAT1 and interleukin 12 (IL-12) after stimulation. Consequently, the diagnosis of AIIA was made. Multidrug therapy was initiated for the concurrent treatment of three simultaneous infections: a) antimycobacterial therapy with azithromycin (500 mg q.d.), rifabutin (300 mg q.d.) and ethambutol (600 mg q.d.), b) virostatic therapy with valganciclovir (450 mg q.d.) and c) antibiotic therapy with cefuroxime (500 mg b.i.d.). Additionally, B-cell-depleting therapy with rituximab (fixed dose protocol: 1000 mg absolute on d1 and d15, followed by 6-monthly continuation therapy; as used for the treatment of other autoantibody-mediated diseases [16]) was initiated after several months of anti-infective therapy as a targeted treatment approach to inhibit further production of IFNγ autoantibodies and was well tolerated. The patient’s general condition gradually improved under therapy and treatment was continued in an outpatient setting. Symptoms improved continuously and a normalization in inflammatory laboratory markers was observed. Follow-up imaging showed stable findings regarding thoracic lymphadenopathy, while blood CMV-PCR remained negative without ongoing ganciclovir continuation therapy.

## Discussion & Conclusions

IFNγ/IL-12 pathway signalling, predominantly activating monocytes, is crucial to control mycobacteria and other intracellular pathogens [1, 17]. Neutralizing anti-IFNγ autoantibodies mimic congenital defects of this pathway and have been identified as a rare cause of an immunodeficiency associated with disseminated NTM disease. Predominantly occurring in patients aged 30 to 50 years of Asian origin, a strong association of anti-IFNγ autoantibodies to certain human leukocyte antigen (HLA) class II molecules has been found [18, 19]. The etiology of anti-IFNγ autoantibody formation remains unclear and in addition to a failure of self-tolerance, cross-reactivity reactions between infectious agents and host proteins have been discussed [8, 9]. The case presented here is remarkable in various ways: First and in contrast to the vast majority of reported cases, our patient presented not solely with a disseminated NTM infection, but with simultaneous systemic infections involving viral, bacterial and mycobacterial pathogens. Second, a *non-typhoidal salmonella* blood stream infection was present, which is very rare in HIV-uninfected patients [20, 21] and has rarely been reported in the context of AIIA. Third, the case demonstrates how diagnosis can be challenging and is easily missed if not considered. While the exact prevalence of AIIA is unknown, recent reports show that anti-IFNγ autoantibodies are detectable in up to 90% of otherwise healthy patients with disseminated NTM [22, 23]. AIIA should therefore always be considered in non-immunocompromised adult patients with recurrent or resistant NTM infections, particularly if other conditions associated with an impaired cellular immune response such as HIV infections and hematological malignancies were ruled out [24]. Further, a slow and incomplete response to specific antimicrobial therapy can be the first hint of this rare condition. Proper diagnosis was delayed in our patient as the inadequate response to treatment was first attributed to a presumed infection of siliconomas following breast implant rupture. It has to be considered however that NTM infections of breast implants have also been reported in non-immunocompromised patients [25, 26]. The unusual histological findings in our case are of particular interest as they directly reflect the underlying pathomechanism. While granuloma formation was seen, suggesting that the Th1 response was not fully suppressed, these were immature, and the surrounding inflammatory reaction was considerably reduced. Matching this observation, patients with congenital defect of the IFNγ/IL-12 signaling axis typically show immature and poorly differentiated, pathogen-rich, lepromatoid granulomas [27, 28]. So far, there is no standardized approach for the treatment of patients with AIIA. While the need for anti-infective therapy stands to reason, the recurrent nature and severity of NTM infections in these patients suggest that treatment of the underlying condition is necessary to achieve long-term control of infections without the need for ongoing anti-infective therapy [2, 29]. B-cell-depleting therapy with rituximab allows for a targeted approach to eliminate anti-IFNγ autoantibody production. While immunosuppressive therapy at first seems counterintuitive in patients with disseminated infections, rituximab has shown promising results in a small study of AIIA patients [29]. Rituximab therapy was well tolerated in our patient who remains in a good overall condition under ongoing anti-mycobacterial treatment at the last outpatient visit 12 months after diagnosis.

Neutralizing autoantibodies against IFNγ can cause an acquired immunodeficiency syndrome characterized by disseminated infections with intracellular pathogens. The disease mainly affects patients of Asian origin and is strongly associated with certain HLA class II allotypes. Since clinical presentation can be highly variable, the diagnosis should be considered in any patient with unexplained opportunistic infections with NTM and other intracellular pathogens, as B-cell-depleting therapy offers a targeted treatment approach for affected patients.

## Data Availability

All data generated or analyzed during this study is included in this published article.

## Abbreviations

AIIA: Adult-onset immunodeficiency with anti-interferon gamma autoantibodies
CMV: cytomegalovirus
CT: Computed tomography
ESR: Erythrocyte sedation rate
IFNγ: Interferon gamma
IL12: Interleukin 12
HIV: Human immunodeficiency virus
HLA: Human leukocyte antigen
NTM: Non-tuberculous mycobacteria
PCR: Polymerase chain reaction
PET: Positron emission tomography
VZV: Varicella zoster virus
